# COVID-19-Induced Refractory Symptomatic Hypocalcemia in a Patient With Parathyroid Gland Reimplantation

**DOI:** 10.1155/2024/6375828

**Published:** 2024-09-24

**Authors:** Crystal Tse, Ho-Man Yeung

**Affiliations:** Department of Medicine Lewis Katz School of Medicine Temple University, Philadelphia, Pennsylvania, USA

**Keywords:** COVID-19, hypocalcemia, parathyroid hormone interference

## Abstract

**Background:** Several cases of severe hypocalcemia in the setting of COVID-19 have been reported. The proposed mechanisms include direct viral interaction with ACE2 receptors in the parathyroid gland, viral chelation of calcium, worsening hypovitaminosis D, critical illness leading to unbound fatty acids binding calcium, and inflammatory cytokines leading to PTH resistance. Given the life-threatening nature of hypocalcemia, this underrecognized phenomenon should be on the forefront of the clinician's attention. This case highlights a rare manifestation of COVID-19 and further complicated by the patient's reimplanted parathyroid gland.

**Presentation:** A 73-year-old female with primary hyperparathyroidism status post parathyroidectomy with reimplantation in the left forearm presented with 4 days of viral syndrome, found to have tetany and Chvostek's sign on physical exam. Pertinent laboratory abnormalities included calcium 5.3 mg/dL, ionized calcium 0.44 mmol/L, magnesium 1.4 mg/dL, phosphorous 5.5 mg/dL, PTH 242 pg/mL, and 25-OH vitamin D 56 ng/mL. Chest CT revealed multifocal pneumonia consistent with positive COVID-19 testing. She was subsequently admitted to the ICU for severe, symptomatic hypocalcemia and was initiated on a continuous calcium infusion, remdesivir, baricitinib, and steroids. Tetany resolved after 9 g calcium repletion, and she was transferred to the medical floor with an ionized calcium of 0.83 mmol/L. On hospital day 3, repeat ionized calcium was 0.78 mmol/L despite ongoing repletion. Given the persistence of hypocalcemia, a repeat PTH level was obtained which remained high at 487 pg/mL, suggesting ongoing PTH interference in the setting of COVID-19. PTH was obtained from the right (nonimplanted) arm which was normal at 74 pg/mL. This indicated an appropriate PTH response from the reimplanted gland, and that ongoing hypocalcemia may be due to insufficient PTH function to maintain systemic calcium levels or a peripheral interference with PTH level. With continued calcium supplementation and treatment of COVID-19, the patient's calcium stabilized at 8.6 mg/dL. She was discharged on oral calcium supplementation with endocrinology follow-up.

**Conclusion:** Acute hypocalcemia strongly correlates with a profound inflammatory response in COVID-19 patients. This case corroborates the cytokine/PTH hypothesis. This patient had a high PTH sampled near the reimplanted gland but an inappropriately normal PTH from the nonimplanted arm, indicating that direct viral interaction interfering with PTH release is an unlikely mechanism. This case represents a scenario where PTH can be sampled directly from the source and this type of model could aid in the process of determining the etiology of hypocalcemia in COVID-19.

## 1. Background

Hypocalcemia is a common abnormality seen in patients with COVID-19 [1]. Recent studies suggest that hypocalcemia may be a distinct feature of hospitalized COVID-19 patients [2]. Higher rates of hypocalcemia in patients are frequently seen with severe acute respiratory infections due to COVID-19 compared to patients with non-COVID-19-related upper respiratory infections [3, 4]. Hypocalcemia is associated with mortality in hospitalized patients [5, 6]. There are various proposed mechanisms underlying low calcium in these patients, including viral chelation of calcium ions, exacerbation of vitamin D deficiency [7], unbound fatty acids in critical illness binding to calcium ions and albumin, and inflammatory cytokines associated with parathyroid hormone (PTH) resistance [8–12].

The hallmarks of acute hypocalcemia include tetany, prolongation of QT intervals, and positive physical exam findings, such as Trousseau's sign and Chvotek's sign. More severe manifestations include seizures and cardiac arrhythmias, such as torsades de pointes. Given the possible life-threatening nature of hypocalcemia, this underrecognized phenomenon should be on the forefront of the clinician's attention.

This case report expands our understanding of COVID-19-induced hypocalcemia as this patient has an implanted parathyroid gland. This report demonstrates elevated PTH near the reimplanted gland but inappropriately low PTH in the peripheral circulation, in the setting of COVID-19-induced hypocalcemia, and delineates a scenario where PTH level can be sampled directly from the source.

## 2. Case Presentation

A 73-year-old female presented to the hospital with dyspnea, fevers, and chills. She was diagnosed with COVID-19, which was complicated by symptomatic hypocalcemia. She was found to have an albumin-corrected calcium level of 6.03 mg/dL (normal laboratory range 8.8–10.2 mg/dL). Vital signs on presentation were as follows: temperature, 98.3°F (36.8°C); heart rate, 76 bpm; blood pressure, 133/85 mmHg; and respiratory rate, 20 breaths per minute, with an O_2_ saturation of 96% on room air.

Her past medical history was notable for primary hyperparathyroidism treated with parathyroidectomy with reimplantation in the left forearm and chronic kidney disease stage 3, with baseline eGFR ~ 41 mL/min/1.73 m^2^. She was not taking calcium or vitamin D supplementation.

**Figure 1:**
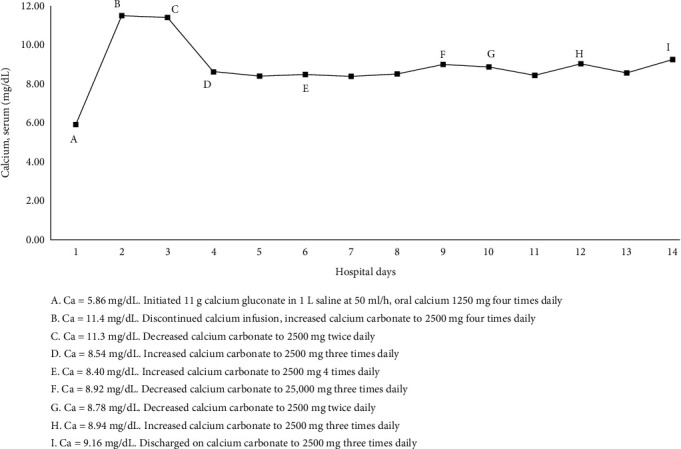
Albumin-adjusted serum calcium levels and interventions plotted over time.

Physical examination was remarkable for positive Chvostek sign and marked tetany in her extremities. Electrocardiogram obtained on admission showed normal sinus rhythm at 82 bpm with nonspecific T-wave flattening and T-wave inversion of the lateral leads with prolonged QTc (567 ms). As shown in Table 1, initial laboratory findings revealed severe hypocalcemia (5.4 mg/dL) (8.8–10.2 mg/dL), with an albumin-corrected calcium level of 6.03 mg/dL (8.9–10.3 mg/dL), low ionized calcium (0.44 mmol/L) (1.0–1.30 mmol/L), elevated PTH (242 pg/mL) (19–88 pg/mL), and hyperphosphatemia (5.5 mg/dL) (2.5–4.9 mg/dL) but normal vitamin D-25 level (56 ng/mL) (>30 ng/mL). The patient's calcium levels and treatment are outlined in Figure 1.

The patient was tested positive for SARS-COV-2 by conventional RT-PCR assay, with multifocal lung opacities. For COVID-19, the patient was treated with baricitinib, remdesivir, and methylprednisolone.

Due to the patient's presentation of symptomatic hypocalcemia and prolonged QTc, the patient was transferred to the ICU for close monitoring. Calcium replacement was initiated with 11 g calcium gluconate in 1 L NS at 50 mL/h and oral carbonate calcium 1250 mg every 4 h. After 6 h on calcium gluconate infusion, calcium improved to 6.1 mg/dL (corrected calcium 6.9). On hospital day 1, calcium gluconate infusion was discontinued. She remained on oral calcium carbonate 2500 mg every 4 h. Physical exam findings improved and was negative for Chvostek sign, and electrocardiogram showed improved QTc of 471 ms. Over the next 3 days, there were slight fluctuations in calcium levels, with an overall downward trend. On hospital day 5, ionized calcium remained low but improved (0.83 mmol/L). Calcium replacement was subsequently adjusted to 2500 mg four times a day.

**Figure 2:**
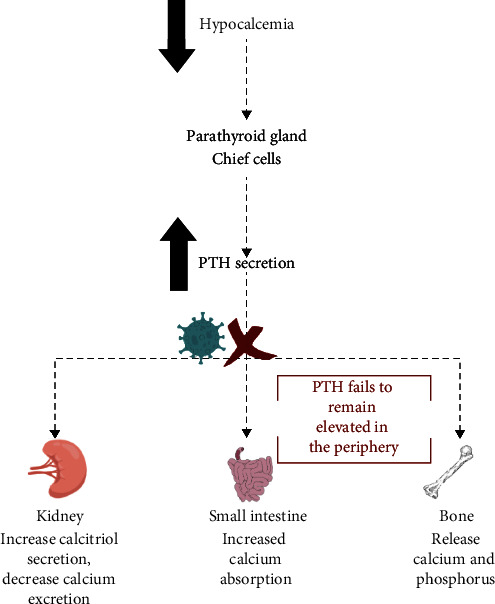
Parathyroid response to hypocalcemia. In response to low calcium levels, the parathyroid gland increases parathyroid hormone (PTH) production. PTH then acts on several organs to increase total serum calcium. However, in this case, PTH level collected near the reimplanted gland was elevated, but it was not sustained in the periphery.

Her venous blood sampling has been from the left upper extremity, which is proximal to the patient's implanted parathyroid gland. Repeat blood tests were then obtained from the contralateral limb (right arm), distal from the parathyroid implant. PTH returned inappropriately normal (74 pg/mL) in the context of low calcium. This finding suggests an appropriate PTH response from the reimplanted gland, and that ongoing hypocalcemia may be due to insufficient PTH function to maintain systemic calcium levels or a peripheral interference with PTH level (Figure 2).

She was discharged in stable condition with prescriptions for calcium carbonate 1250 mg three times daily. Unfortunately, the patient was lost to follow-up with outpatient endocrinology.

## 3. Discussion

This case highlights a rare manifestation of COVID-19 that is further complicated by the patient's reimplanted parathyroid gland. Other possible causes of hypoparathyroidism do not explain this patient's hypocalcemia. The patient had a history of hyperparathyroidism and underwent a parathyroidectomy 6 years prior to her admission. Patients who undergo a parathyroidectomy may experience hypoparathyroidism or vitamin D deficiency. The patient also received remdesivir and methylprednisolone for treatment of COVID-19. Methylprednisolone, a glucocorticoid, is most notably known to cause hypocalcemia in patients with hypoparathyroidism by reducing calcium reabsorption in the intestine [13]. However, on initial presentation, this patient had an elevated PTH of 242 pg/mL and a normal vitamin D level of 56 ng/mL, which suggests that the parathyroidectomy was not a probable cause of her hypocalcemia. Remdesivir has also been reported to cause hypocalcemia. One retrospective observational study found that remdesivir was associated with hypocalcemia in more than 10% of patients who received treatment. However, the study did not have a control group which made it difficult to distinguish whether the metabolic abnormalities were due to remdesivir, COVID-19, or another underlying disease [14]. In contrasts, several cases reports noted hypocalcemia in patients with COVID-19 who did not receive remdesivir for treatment [15, 16]. Together, this suggests that the treatment targeting COVID-19 was not responsible for low calcium levels.

Additionally, this patient has a history of CKD stage 3, a well-known cause of secondary hyperparathyroidism, which is characterized by elevated PTH, low calcium, and high phosphorus. PTH stimulates renal calcitriol (1,25D3) synthesis, which promotes reabsorption of calcium and phosphorous, but patients with chronic kidney disease have a reduced ability to produce calcitriol. Thus, patients with chronic kidney disease produce higher levels of PTH to compensate for the reduction in calcium reabsorption [17]. Secondary hyperparathyroidism is also associated with low bone mineral density and elevated alkaline phosphatase. In contrast, this patient presented with normal vitamin D levels (56.0 ng/mL) and normal alkaline phosphatase (66.0 U/L), and her most recent DEXA scan from 2 years prior showed no evidence of reduced bone mineral density. Given these findings, secondary hyperparathyroidism is less likely the cause of her hypocalcemia.

Prior studies proposed several possible mechanisms for the reduced compensatory PTH response seen in patients with COVID-19. Studies conducted on the previous generation of coronavirus (SARS-CoV), which was responsible for the severe acute respiratory syndrome (SARS) epidemic in 2003, showed that patients who died of SARS had SARS-CoV RNA and antigenic material in their parathyroid gland cells [18]. In addition, SARS-CoV was associated with increased ACE2 receptors on parathyroid glands [19]. This suggests that SARS-CoV-2 may potentially directly invade parathyroid glands. In contrast, numerous animal studies show cytokine-induced hypocalcemia in association with upregulated calcium-sending receptor expression in the kidney and parathyroid [20]. Lastly, several studies suggest that COVID-19 is associated with impaired PTH secretion, especially in the context of vitamin D deficiency [1, 21].

For the normal physiology of calcium homeostasis, calcium sensing receptors sense alterations to serum calcium levels and help to normalize its concentration by regulating PTH secretion. In response to hypocalcemia, the parathyroid gland upregulates PTH levels. PTH in the periphery stimulates calcium and phosphate release from bone, calcium reabsorption from the renal distal tubule, and renal production of calcitriol, which encourages renal and intestinal reabsorption of both calcium and phosphate [22]. However, this patient had an elevated PTH level sampled near the reimplanted gland but an inappropriately normal PTH when sampled distal from the reimplanted gland, indicating that direct viral interaction interfering with PTH release is an unlikely mechanism. This supports that PTH released by the parathyroid gland does not sustain in peripheral circulation, leading to decompensated hypocalcemia. The impaired PTH response to hypocalcemia in COVID-19 is yet to be understood. One limitation in this case is that the PTH levels were obtained sequentially rather than simultaneously from both the implanted and nonimplanted arms. The PTH ratio between the implanted and the nonimplanted arm greater than 1.5 would indicate appropriate gland function [23], which would further support the hypothesis that PTH interference occurs in the periphery. Based on the PTH trend, we would expect the PTH ratio to be greater than 1.5, though simultaneous sampling was not performed in real-time practice to eliminate spurious PTH elevation as a possibility.

## 4. Conclusion

To date, there are no cases that discuss COVID-19-induced hypocalcemia in the setting of parathyroid reimplantation. This case represents a scenario where PTH can be sampled directly from the source and is a model that could aid in the process of determining the etiology of hypocalcemia in COVID-19. This case also highlights the importance of careful surveillance of hypocalcemia in patients with COVID-19. Given the dangerous nature of acute hypocalcemia, we suggest monitoring calcium levels in patients hospitalized with COVID-19 infection.

## Figures and Tables

**Table 1:** Laboratory tests on admission, day 5, day 7, and day 14 or day of discharge.

Tests	Day 1	Day 5	Day 7	Day 14
Total calcium (8.8–10.2 mg/dL)	5.30 (L)	7.60 (L)	7.60 (L)	8.6 (L)
Calcium, ion (1.0–1.30 mmol/L)	0.44 (L)	0.83 (L)	0.84 (L)	—
Albumin-corrected calcium	5.86 (L)	8.32 (L)	8.32 (L)	9.16 (N)
Albumin (3.2–4.6 g/dL)	3.30 (N)	3.10 (L)	3.10 (L)	3.30 (N)
PTH (19–88 pg/mL)	242 (H)	487 (H)	74.0 (N) (collected from R arm)	—
Vitamin D-25 (>30 ng/mL)	56.0 (N)	—	—	—
Phosphorous (2.5–4.9 mg/dL)	5.50 (H)	4.20 (N)	3.50 (N)	4.40 (N)
Magnesium (1.6–2.4 mg/dL)	1.40 (L)	2.00 (N)	2.00 (N)	2.10 (N)
Alkaline phosphatase (45–117 U/L)	66.0 (N)	59.0 (N)	57.0 (N)	63.0 (N)
eGFR (>60 mL/min/1.73 m^2^)	41.0 (L)	>60 (N)	>60 (N)	54.0 (N)
D-dimer (0–500 ng/mL)	1,283 (H)	762 (H)	734 (H)	807 (H)
CRP (0.0–0.4 mg/dL)	11.0 (H)	2.40 (H)	1.20 (H)	2.60 (H)

## Data Availability

Data sharing is not applicable to this article as no new data were created or analyzed in this study.
